# Risk factors for respiratory complications after adenotonsillectomy in children with obstructive sleep apnea*

**DOI:** 10.1590/S1806-37132015000004415

**Published:** 2015

**Authors:** Renato Oliveira Martins, Nuria Castello-Branco, Jefferson Luis de Barros, Silke Anna Theresa Weber

**Affiliations:** 1. Master's Student in Fundamentals of Surgery. Faculdade de Medicina de Botucatu, Universidade Estadual Paulista - FMB-UNESP, São Paulo State University Botucatu School of Medicine - Botucatu, Brazil; 2. Postdoctoral Student in Sleep Medicine. Faculdade de Medicina da Universidade de São Paulo - FMUSP, University of São Paulo School of Medicine - São Paulo, Brazil; 3. Tenured Professor. Department of Ophthalmology, Otolaryngology and Head and Neck Surgery, Faculdade de Medicina de Botucatu, Universidade Estadual Paulista - FMB-UNESP, São Paulo State University Botucatu School of Medicine - Botucatu, Brazil

**Keywords:** Postoperative complications, Tonsillectomy, Sleep apnea, obstructive

## Abstract

**OBJECTIVE:**

To identify risk factors for respiratory complications after adenotonsillectomy in children ≤ 12 years of age with obstructive sleep apnea who were referred to the pediatric ICU (PICU).

**METHODS:**

A cross-sectional historical cohort study analyzing 53 children after adenotonsillectomy who met predetermined criteria for PICU referral in a tertiary level teaching hospital. The Student's t-test, Mann-Whitney test, and chi-square test were used to identify risk factors.

**RESULTS:**

Of the 805 children undergoing adenotonsillectomy between January of 2006 and December of 2012 in the teaching hospital, 53 were referred to the PICU. Twenty-one children (2.6% of all those undergoing adenotonsillectomy and 39.6% of those who were referred to the PICU) had respiratory complications. Of those 21, 12 were male. The mean age was 5.3 ± 2.6 years. A high apnea-hypopnea index (AHI; p = 0.0269), a high oxygen desaturation index (ODI; p = 0.0082), a low SpO_2_ nadir (p = 0.0055), prolonged orotracheal intubation (p = 0.0011), and rhinitis (p = 0.0426) were found to be independent predictors of respiratory complications. Some of the complications observed were minor (SpO_2_ 90-80%), whereas others were major (SpO_2_ ≤ 80%, laryngospasm, bronchospasm, acute pulmonary edema, pneumonia, and apnea).

**CONCLUSIONS:**

Among children up to 12 years of age with OSA, those who have a high AHI, a high ODI, a low SpO_2_ nadir, or rhinitis are more likely to develop respiratory complications after adenotonsillectomy than are those without such characteristics.

## Introduction

Obstructive sleep apnea (OSA) affects approximately 4% of the pediatric population^(^ 1 ^)^ and is associated with significant medical problems, including cardiopulmonary abnormalities^(^ 2 ^,^ 3 ^)^ and failure to thrive.^(^ 4 ^)^

OSA is characterized by increased upper airway resistance leading to disordered sleep. Adenotonsillectomy has become the most common approach in the treatment of pediatric OSA, increasing from 0% in 1978^(^ 5 ^)^ to approximately 77% in 2005,^(^ 6 ^)^ because it significantly improves disordered sleep, physical and emotional symptoms,^(^ 7 ^)^ and systemic inflammation secondary to OSA,^(^ 2 ^)^ and because it can reverse cor pulmonale. ^(^ 3 ^)^

Adenotonsillectomy is not risk-free; there is the possibility of hemorrhage, dehydration, nausea, vomiting, pain,^(^ 8 ^)^ and need for additional airway support postoperatively.^(^ 9 ^-^ 11 ^)^ In children undergoing adenotonsillectomy for OSA, the rate of respiratory complications requiring medical intervention ranges from 21% to 36%.^(^ 9 ^,^ 10 ^)^ OSA is commonly associated with risk factors for respiratory complications. It is important to identify those factors that can increase the risk of postoperative respiratory complications in children with OSA to ensure better quality of care and for safety reasons.

There is a consensus that children with severe OSA should be observed postoperatively.^(^ 1 ^)^ However, there is disagreement over the safest place for clinical observation after surgery: an outpatient setting; a pediatric ward; or a pediatric ICU (PICU). Because of the paucity of evidence-based guidelines and studies for enabling better clinical practice, the objective of this study was to identify risk factors for possible respiratory complications after adenotonsillectomy in children ≤ 12 years of age with OSA who were referred to the PICU.

## Methods

This was a cross-sectional historical cohort study analyzing 53 children after adenotonsillectomy who met predetermined criteria for PICU referral in a tertiary level teaching hospital.

This study was approved by the Research Ethics Committee of the São Paulo State University Botucatu School of Medicine (Protocol no. CEP 4336-2012).

We included all male and female children 1 to 12 years of age, with adenotonsillar hypertrophy, admitted to the PICU after adenotonsillectomy, between January of 2006 and December of 2012. We excluded children with cardiac, pulmonary, neuromuscular, or chromosomal abnormalities and children with craniofacial anomalies, as well as children concurrently undergoing other surgical procedures associated with adenotonsillectomy, such as myringotomy, insertion of ventilation tubes, and/or diagnostic laryngoscopy. Referral to the ICU was based on clinical and/or polysomnographic criteria, which included age < 3 years, obesity, underweight, asthma, and/or polysomnographic changes (SpO_2_ nadir ≤ 80%, AHI ≥ 10 events/h).

All patients were evaluated by a single researcher during the perioperative period. Preoperative data were collected with a standardized history-taking questionnaire regarding age, gender, body mass index (BMI), presence of comorbidities-obesity (as defined on the basis of age-specific and gender-specific BMI percentile curves adopted by the World Health Organization [2007], with BMI percentiles ≥ 97 being a criterion of obesity), asthma, rhinitis, and upper respiratory tract infection (URTI)-adenoid size (as determined by examination with a 2.4-mm-diameter rigid endoscope with a 0-degree lens, on the basis of the adenoid/nasopharyngeal ratio), and tonsil size (as measured by Brodsky's scale)

Cardiorespiratory monitoring to confirm the diagnosis of OSA was performed on an inpatient basis, through the Department of Otolaryngology, for up to 6 months before adenotonsillectomy. The children underwent type I polysomnography (Alice^(r)^; Phillips Respironics, Murrysville, PA, USA), or type III polysomnography (Stardust II^(r)^; Phillips Respironics), or overnight oximetry to record SpO_2_ (PV 4000 LCD; Protec Equipamentos Médico-Hospitalares, São Paulo, Brazil). The respiratory parameters analyzed were as follows: apnea-hypopnea index (AHI); hypopnea index (HI); oxygen desaturation index (ODI); and SpO_2_ nadir. Data were recorded by the software of each device and were scored by a single rater. Oximetry was used to determine SpO_2_ nadir. Obstructive apnea was defined as a greater than 90% drop in nasal pressure excursions for at least 2 respiratory cycles, associated with thoracic and/or abdominal effort. Central apnea was defined as an absence of inspiratory effort throughout the event, with the event being ≥ 20 seconds in duration or lasting 2 respiratory cycles and being associated with an arousal or ≥ 3% oxygen desaturation. Hypopnea was characterized by a decrease of at least 50% in nasal pressure excursions, associated with ≥ 3% oxygen desaturation. The ODI was defined as the number of episodes of oxyhemoglobin desaturation ≥ 3% from baseline SpO_2_ per hour of sleep and ≥ 10 seconds in duration. SpO_2_ nadir was defined as the lowest SpO_2_ value, regardless of duration. OSA was classified as mild (AHI of 1 to 4.9 events/h), moderate (AHI of 5 to 9.9 events/h), or severe (AHI ≥ 10 events/h), and events were scored according to the recommended rules by the American Academy of Sleep Medicine (2007).

All surgical procedures were supervised, which allowed standardization of the surgical technique and the use of a standardized anesthetic protocol for children with OSA. Tonsillectomies and adenoidectomies were performed with the standard cold technique and a combination of intravenous (propofol [3mg/kg] and alfentanil [50mg/kg]) and inhalational (sevoflurane and/or N_2_O/O_2_ 50:50) anesthesia. After surgery, all children were referred, intubated, to the PICU, where they remained intubated for up to 6 h and where they stayed for a minimum of 24 h, in accordance with the guidelines of the facility for children with severe apnea. The children were divided into two groups on the basis of absence of presence of respiratory complications after adenotonsillectomy.

Postoperative respiratory complications were divided into major complications (SpO_2_ ≤ 80%, laryngospasm, bronchospasm, apnea, pneumonia [confirmed by chest X-ray, leukocytosis with a left shift, and fever], and post-obstructive acute pulmonary edema [confirmed by chest X-ray or use of loop diuretics and reintubation]) and minor complications (SpO_2_between 90% and 80% requiring airway repositioning). In addition, we assessed duration of orotracheal intubation (OTI) after adenotonsillectomy as well as medical interventions (need for airway repositioning, use of positive pressure devices [continuous positive airway pressure or bilevel positive airway pressure], and reintubation).

### Statistical analysis

Normality of data was tested with the Kolmogorov-Smirnov test, which was applied to all continuous variables in each group. The Student's t-test, Mann-Whitney test, and chi-square test were used for between-group comparisons of the study variables. Multiple linear regression analysis was performed to determine which variables correlated most closely with an increased risk of respiratory complications after adenotonsillectomy.

All tests were performed with Statistica, version 6.0 (StatSoft Inc., Tulsa, OK, USA), and the level of significance was set at 5%.

## Results

Between January of 2006 and December of 2012, 805 adenotonsillectomies were performed in children with OSA in the teaching hospital, and 53 of those children were referred to the PICU. The reasons for referral to the PICU were age < 2 years (n = 2); obesity and/or asthma with an SpO_2_ nadir ≤ 80% (n = 10); an SpO_2_ nadir ≤ 75% (n = 4); mild OSA with an SpO_2_ nadir ≤ 70% (n = 1); moderate OSA associated with comorbidities (n = 5) or an SpO_2_ nadir ≤ 80% (n = 2); and severe OSA (n = 29). Respiratory parameters were recorded by type I polysomnography (n = 7), type III polysomnography (n = 30), and oximetry (n = 6).

Among the comorbidities assessed, rhinitis was the most common, occurring more frequently in the children with respiratory complications than in those without (Table 1). The children with respiratory complications had a higher AHI, a higher ODI, a lower SpO_2_ nadir, and a longer duration OTI than did those without complications (Table 2). After multiple linear regression analysis, the following independent variables were found to contribute to increasing the risk of respiratory complications after adenotonsillectomy: AHI; ODI; SpO_2_ nadir; rhinitis; and duration of OTI [p(R^2)^ = 0.0099]; although, individually, no variable showed a close association with the clinical outcome (Table 3).

**Table 1.** Comparison of demographic data and comorbidities in children, by absence or presence of respiratory complications after adenotonsillectomy.a

Variable	Respiratory complications after adenotonsillectomy		p
	Absence	Presence	
	(n = 32)	(n = 21)	
Male/Female	16/16	12/9	0.6062*
Age, years	6.2 ± 3.1	5.3 ± 2.6	0.2820**
< 3	4	3	0.4040***
3-6	13	10	
7-9	8	7	
10-12	7	1	
BMI, kg/m^2^	19.28 ± 5.27	18.76 ± 5.19	0.7257**
< 3rd percentile	0	2	0.8489***
≥ 3rd percentile and < 85th percentile	14	7	
≥ 85th percentile and < 97th percentile	5	2	
≥ 97th percentile	13	10	
Comorbidity			
Obesity	13	10	0.4931*
Asthma	4	6	0.1164*
Rhinitis^b^	20	25	0.0426*
Current URTI	0	2	0.0668*

BMI: body mass index; and URTI: upper respiratory tract infection. aValues expressed as n of patients or as mean ± SD. bVariable selected for multiple linear regression analysis.

* Chi-square test;

** Student's t-test; and

*** Mann-Whitney test.

**Table 2.** Comparison of polysomnographic data, duration of orotracheal intubation, and adenoid and tonsil size in children, by absence or presence of respiratory complications after adenotonsillectomy.

Variable	Absence of complications		Presence of complications		p
	n of patients	Mean ± SD	n of patients	Mean ± SD	
Polysomnographic variable					
AHI, events/h^a^	24	18.1 ± 11.2	13	28.6 ± 16.3	0.0269*
HI, events/h	24	5.8 ± 6.0	13	10.5 ± 10.4	0.0882*
ODI, episodes/h^a^	18	15.3 ± 9.4	12	29.8 ± 18.4	0.0082*
SpO_2_ nadir, %^a^	28	76.8 ± 10.9	15	64.4 ± 16.9	0.0055*
PO duration of OTI, h^a^	32	1.8 ± 2.0	21	5.0 ± 4.8	0.0011*
Adenoid and tonsil size					
Adenoid, ANR	30	78 ± 17	17	87 ± 13	0.0705*
Tonsils, Brodsky’s scale					
1	0	−	0	−	0.3512**
2	4	−	2	−	
3	18	−	10	−	
4	10	−	9	−	

AIH: apnea-hypopnea index; HI: hypopnea index; ODI: oxygen desaturation index; PO: postoperative; OTI: orotracheal intubation; and ANR: adenoid/nasopharyngeal ratio. aVariables selected for multiple linear regression analysis.

* Student's t-test; and

** Mann-Whitney test.

**Table 3.** Multiple linear regression analysis considering respiratory complications after adenotonsillectomy as a dependent variable.a

Independent variable	Absence of complications	Presence of complications	Beta	Partial regression coefficient	Standard error	p	R^2^	p (R^2^)
AHI	18.1 ± 11.2	28.6 ± 16.3	−0.0276	−0.0009	0.0073	0.8972	0.3722	< 0.0099
ODI	15.3 ± 9.4	29.8 ± 18.4	0.2730	0.0082	0.0075	0.2798		
SpO_2_ nadir	76.8 ± 10.9	64.4 ± 16.9	−0.0077	−0.0003	0.0062	0.9650		
Rhinitis	20	25	0.2679	0.3265	0.1843	0.0863		
PO duration of OTI, h	1.8 ± 2.0	5.0 ± 4.8	0.3781	0.0527	0.0267	0.0568		

AIH: apnea-hypopnea index; ODI: oxygen desaturation index; PO: postoperative; and OTI: orotracheal intubation. aValues expressed as mean ± SD or as n of patients.

Of the 53 children studied, 21 (39.6%) had respiratory complications after adenotonsillectomy. The children were divided into two groups on the basis of absence of presence of respiratory complications (Table 1). The group without complications consisted of 32 children, 16 of whom were male, and the mean age was 6.1 ± 3.1 years (range, 1.6-12 years). The group with complications consisted of 21 children, 12 of whom were male, and the mean age was 5.3 ± 2.6 years (range, 2.4-12 years).

Seven children had minor respiratory complications (SpO_2_ 90-80%), and 14 children had major respiratory complications (SpO_2_ ≤ 80% [n = 2]; laryngospasm [n = 9]; bronchospasm [n = 5]; intraoperative bronchospasm [n = 2]; apnea [n = 1]; pneumonia [n = 1]; and acute pulmonary edema [n = 3]; Table 4). The group with respiratory complications remained in the PICU for ≥ 24 h, and the main medical interventions were antibiotic therapy (n = 1) for pneumonia, use of loop diuretics (n = 3) for acute pulmonary edema, continuous administration of nebulized bronchodilator or adrenaline (n = 12) for bronchospasm and laryngospasm, and reintubation (n = 3) for acute pulmonary edema and severe bronchospasm in the presence of URTI (Table 4). The postoperative mortality rate was zero, and the children with and without respiratory complications remained hospitalized for 3 ± 1 days and 5 ± 2 days, respectively.

**Table 4.** Individual, descriptive data of children with major or minor respiratory complications after adenotonsillectomy.

Patient	Gender	Age (years)	BMI percentile	Postoperative complication	Reintubation	AHI	HI	ODI	< SpO_2_ (%)	Comorbidity
(events/h)	(events/h)	(episodes/h)
Major respiratory complication										
1	M	7.0	85-97	APE	Yes	8.8	5.3	18.2	80	Asthma + rhinitis
2	M	3.6	≥ 97	APE + laryngospasm	Yes	*	*	*	*	Asthma + obesity + rhinitis
3	M	3.3	≥ 97	APE + laryngospasm	No	*	*	*	*	Obesity + rhinitis
4	F	7.1	≥ 97	Laryngospasm	No	17.8	11.7	25.6	82	Obesity + rhinitis
5	F	4.7	85-97	Laryngospasm	No	46.4	19	40.2	70	Rhinitis
6	M	7.7	≥ 97	Laryngospasm	No	*	*	*	*	Obesity + rhinitis
7	M	2.6	50-85	Laryngospasm + pneumonia	No	*	*	*	42	URTI+ rhinitis
8	M	2.7	50-85	Laryngospasm + bronchospasm	Yes	*	*	*	72	URTI + rhinitis
9	F	2.4	50-85	Laryngospasm + intraoperative and postoperative bronchospasm	No	*	*	*	*	Asthma + rhinitis
10	F	3.3	50-85	Laryngospasm + bronchospasm	No	18.7	7.2	16.6	55	Rhinitis
11	F	4.1	≥ 97	Intraoperative bronchospasm + SpO_2_≤ 80%	No	*	*	*	*	Obesity + rhinitis
12	M	3.2	50-85	Bronchospasm + apnea	No	33.1	2	30.8	63	Rhinitis
13	M	3.6	≥ 97	Bronchospasm	No	11.4	6.7	11.3	41	Asthma + obesity + rhinitis
14	F	3.6	15-50	SpO_2_ ≤ 80%	No	*	*	*	*	Asthma + rhinitis
Minor respiratory complication										
1	M	8.0	≤ 3	SpO_2_ 90-80%	No	18.9	5.8	0	70	Asthma + rhinitis
2	M	3.7	50-85	SpO_2_ 90-80%	No	26.1	4.5	36.6	46	Asthma + rhinitis
3	M	4.7	≥ 97	SpO_2_ 90-80%	No	33.2	13.6	55.2	51	Obesity
4	F	7.4	≥ 97	SpO_2_ 90-80%	No	55.8	40.3	67.7	58	Obesity + rhinitis
5	M	12.0	≤ 3	SpO_2_ 90-80%	No	45.4	13.6	29.9	53	Rhinitis
6	F	8.0	≥ 97	SpO_2_ 90-80%	No	48.3	7.2	23.8	88	Obesity + rhinitis
7	F	7.2	≥ 97	SpO_2_ 90-80%	No	8	0	1.9	95	Obesity + rhinitis

BMI: body mass index; AHI: apnea-hypopnea index; HI: hypopnea index; ODI: oxygen desaturation index; M: male; APE: acute pulmonary edema; F: female; and URTI: upper respiratory tract infection.

The respiratory complications observed after adenotonsillectomy are described individually in Table 4.

## Discussion

Of the 805 children undergoing adenotonsillectomy between January of 2006 and December of 2012 in the teaching hospital, 21 (2.6%) had postoperative respiratory complications. This finding is similar to the results of other studies, in which the rate of respiratory complications ranged from 1.3% to 13.4%.^(^ 12 ^-^ 16 ^)^ Analysis of the children at high risk for complications who were referred to the PICU (n = 53) revealed that the rate of respiratory complications was 39.6%, which is also consistent with the findings of other studies, in which rates ranged from 25% to 60%. ^(^ 11 ^,^ 17 ^-^ 21 ^)^ It is of note that none of the children who had post-adenotonsillectomy follow-up in the pediatric ward had any major respiratory complications requiring transfer to the PICU.

The postoperative referral of the 53 children to the PICU was motivated by the presence of OSA associated with one or more risk factors. According to the literature, children with OSA aged < 2 years ^(^ 9 ^,^ 11 ^,^ 17 ^,^ 18 ^)^ or < 3 years^(^ 21 ^)^ and presenting with obesity,^(^ 9 ^,^ 12 ^,^ 15 ^,^ 22 ^-^ 24 ^)^ underweight,^(^ 9 ^,^ 21 ^)^ asthma,^(^ 11 ^,^ 14 ^)^ polysomnographic changes (SpO_2_ nadir ≤ 80%^(^ 11 ^)^ or < 72%,^(^ 9 ^,^ 15 ^,^ 19 ^)^ AHI ≥ 24 events/h,^(^ 17 ^)^ high HI and/or high AHI),^(^ 15 ^)^ CO_2_ pressure > 45 mmHg and SpO_2_ < 86%,^(^ 21 ^)^ intraoperative laryngospasm,^(^ 17 ^)^ or systemic comorbidities (neuromuscular abnormalities,^(^ 9 ^,^ 11 ^,^ 20 ^)^ craniofacial anomalies,^(^ 9 ^,^ 11 ^,^ 20 ^)^ cardiac abnormalities,^(^ 11 ^,^ 12 ^,^ 20 ^,^ 25 ^)^ and chromosomal abnormalities)^(^ 11 ^,^ 12 ^)^ have increased rates of respiratory complications after adenotonsillectomy. However, after this study's analyses of the results for postoperative respiratory complications, the local department of otolaryngology discontinued the routine practice of referring patients < 12 years of age with OSA and/or risk factors to the PICU after adenotonsillectomy. What is recommended is that a sleep study be performed to determine the severity of the respiratory disorder and a thorough clinical history be taken to identify risk factors. For patients with severe OSA associated with severe comorbidities (chromosomal, cardiac, and neuromuscular abnormalities, as well as craniofacial anomalies) and/or children < 2 years of age, the recommendation for postoperative observation in the PICU still applies.

The risk factors that could predict respiratory complications after adenotonsillectomy were polysomnographic parameters (high AHI [mean of 28.6 events/h]; high ODI [mean of 29.8 episodes/h]; and low SpO_2_ nadir [mean of 64.4%]), presence of rhinitis, and prolonged postoperative OTI (Table 2).

A higher AHI, a higher ODI, and a lower SpO_2_ nadir on the preoperative sleep study translate to a higher prevalence of respiratory complications in children. Our results were similar to those found in other studies that correlated polysomnographic findings with postoperative respiratory complications and medical interventions.^(^ 10 ^,^ 11 ^,^ 15 ^,^ 17 ^,^ 26 ^)^ Schroeder et al.^(^ 26 ^)^ observed that 43% of the children with an AHI > 25 events/h required some intervention as a result of respiratory complications. Another study showed that a higher AHI (mean of 31.8 events/h), a higher HI (mean of 22.6 events/h), and a lower SpO_2_ nadir (mean of 71.7%) translate to a stronger correlation with respiratory complications, and that desaturation events were the ones most commonly requiring oxygen supplementation. ^(^ 15 ^)^ Therefore, it is clear that determining apnea severity via a sleep study (polysomnography) before surgery is important in predicting the risk of possible postoperative respiratory complications.

Among the comorbidities assessed, only rhinitis was found to be statistically significant in the group with respiratory complications. Because children with severe OSA have greater airway collapsibility^(^ 27 ^,^ 28 ^)^ and are more susceptible to the respiratory-depressant effects of anesthetics and opioids,^(^ 29 ^,^ 30 ^)^ it is possible that rhinitis associated with increased secretions and upper airway edema can contribute to greater resistance and, consequently, to an increased likelihood of perioperative respiratory complications.

Although several studies in the literature have demonstrated that age < 2 years^(^ 9 ^,^ 11 ^,^ 17 ^,^ 18 ^)^ or < 3 years,^(^ 21 ^)^ obesity,^(^ 9 ^,^ 12 ^,^ 15 ^,^ 22 ^-^ 24 ^)^ and asthma^(^ 11 ^,^ 14 ^)^ are related to a greater likelihood of respiratory complications after adenotonsillectomy, this study found no statistically significant differences among these variables in terms of the occurrence of respiratory events (Table 1). It may be that this lack of significance is due to the small sample size, to the fact that this was a group of children at increased risk for respiratory complications, and to the fact that the children were similar in terms of comorbidities.

Duration of OTI (Table 2) was statistically longer in patients with respiratory complications, corroborating Schroeder Jr. et al.,^(^ 26 ^)^ who demonstrated that delaying extubation can increase the likelihood of respiratory complications in a group who is already at high risk. Therefore, children undergoing adenotonsillectomy for OSA associated with comorbidities who remained intubated after surgery had increased complication rates and, consequently, prolonged hospital stays.

This study was limited by its small sample size, surgeons' varied preferences of where postoperative observation should occur, and a possible bias in selecting children for polysomnography. A prospective study with a larger sample size is needed to determine possible risk factors associated with an increased likelihood of respiratory complications after adenotonsillectomy.^(^ 17 ^)^

The results of the present study indicate that, among children up to 12 years of age diagnosed with OSA, those who have a high AHI, a high ODI, and a low SpO_2_ nadir on preoperative polysomnography, as well as rhinitis, are more likely to develop respiratory complications after adenotonsillectomy than are those without such characteristics.
