# Supplementary material for: Risk factors for respiratory complications after adenotonsillectomy in children with obstructive sleep apnea
Source: J Bras Pneumol. 2015 May-Jun;41(3):238–45. doi: 10.1590/S1806-37132015000004415 (PMC4541759; doi:10.1590/S1806-37132015000004415)
Supplement: Translation

# Translation: Fatores de risco para complicações respiratórias após adenotonsilectomia em crianças com apneia obstrutiva do sono*

**Authors:** Renato Oliveira Martins, Nuria Castello-Branco, Jefferson Luis de Barros, Silke Anna Theresa Weber

**Affiliations:** 1 Mestrando em Bases Gerais da Cirurgia. Faculdade de Medicina de Botucatu, Universidade Estadual Paulista - FMB-UNESP - Botucatu (SP) Brasil; 2 Pós-Doutoranda em Medicina do Sono. Faculdade de Medicina da Universidade de São Paulo - FMUSP - São Paulo (SP) Brasil; 3 Professora Livre Docente. Disciplina de Otorrinolaringologia, Faculdade de Medicina de Botucatu, Universidade Estadual Paulista - FMB-UNESP - Botucatu (SP) Brasil

**Keywords:** Complicações pós-operatórias, Tonsilectomia, Apneia do sono tipo obstrutiva

## Abstract

**OBJETIVO:**

Identificar fatores de risco para complicações respiratórias após adenotonsilectomia em crianças ≤ 12 anos com apneia obstrutiva do sono encaminhadas à UTI pediátrica (UTIP).

**MÉTODOS:**

Estudo de coorte histórica com corte transversal que analisou 53 crianças após adenotonsilectomia que preencheram os critérios pré-estabelecidos para encaminhamento à UTIP em um hospital escola de nível terciário. Foram utilizados o teste t de Student, o teste de Mann-Whitney e o teste do qui-quadrado para identificar os fatores de risco.

**RESULTADOS:**

Das 805 crianças submetidas à adenotonsilectomia entre janeiro de 2006 e dezembro de 2012 no hospital escola, 53 foram encaminhadas à UTIP. Vinte e uma crianças (2,6% do total de submetidas à adenotonsilectomia e 39,6% das que foram encaminhadas à UTIP) apresentaram complicações respiratórias, sendo 12 do gênero masculino e a idade média de 5,3 ± 2,6 anos. Maior índice de apneia-hipopneia (IAH; p = 0,0269), maior índice de dessaturação de oxigênio (IDO; p = 0,0082), baixo nadir da SpO_2_ (p = 0,0055), maior tempo de intubação orotraqueal (p = 0,0011) e rinopatia (p = 0,0426) foram preditores independentes de complicações respiratórias. Foram observadas complicações respiratórias menores (SpO_2_ entre 90-80%) e maiores (SpO_2_ ≤ 80%, laringoespasmos, broncoespasmos, edema agudo de pulmão, pneumonia e apneia).

**CONCLUSÕES:**

Em crianças de até 12 anos e com apneia obstrutiva do sono, aquelas que têm maior IAH, maior IDO, menor nadir da SpO_2_ e/ou rinopatia são mais predispostas a desenvolver complicações respiratórias após adenotonsilectomia do que aquelas sem essas características.

## Introdução

A apneia obstrutiva do sono (AOS) afeta cerca de 4% da população pediátrica^(^ 1 ^)^ e está relacionada com significativos problemas médicos que incluem anormalidades cardiopulmonares^(^ 2 ^,^ 3 ^)^ e insuficiência do crescimento.^(^ 4 ^)^

A AOS é caracterizada por um aumento da resistência das vias aéreas superiores (VAS) que leva ao distúrbio do sono. A adenotonsilectomia tornou-se a indicação mais comum no tratamento da AOS pediátrica, passando de 0% em 1978^(^ 5 ^)^ para aproximadamente 77% em 2005,^(^ 6 ^)^ por melhorar significativamente o distúrbio do sono, os sintomas físicos e emocionais,^(^ 7 ^)^ os distúrbios sistêmicos secundários à AOS^(^ 2 ^)^ e por ser capaz de reverter o cor pulmonale.^(^ 3 ^)^

A adenotonsilectomia não é isenta de riscos e apresenta a possibilidade de hemorragias, desidratação, náuseas, vômitos, dor^(^ 8 ^)^ e necessidade de suporte de via aérea adicional no pós-operatório. ^(^ 9 ^-^ 11 ^)^ Em crianças submetidas à adenotonsilectomia para AOS, a taxa de complicações respiratórias com indicação de intervenção médica variou de 21 a 36%.^(^ 9 ^,^ 10 ^)^ A AOS está frequentemente associada a fatores de risco para complicações respiratórias. É importante determinar quais são esses fatores que podem aumentar o risco de complicações respiratórias pós-operatórias em crianças com AOS para uma melhor qualidade do tratamento e por uma razão de segurança.

Há um consenso de que crianças com AOS grave devem ser observadas durante o período pós-operatório.^(^ 1 ^)^ No entanto, há uma discordância quanto ao local mais seguro para a observação clínica após a cirurgia: ambiente ambulatorial, enfermaria pediátrica ou UTI pediátrica (UTIP). Devido à escassez de diretrizes e estudos baseados em evidências que permitam uma melhor prática clínica, o objetivo deste estudo foi identificar fatores de risco para possíveis complicações respiratórias após adenotonsilectomia em crianças ≤ 12 anos com AOS encaminhadas à UTIP.

## Métodos

Estudo de coorte histórica com corte transversal que analisou 53 crianças após adenotonsilectomia que preencheram os critérios pré-estabelecidos para encaminhamento à UTIP em um hospital escola de nível terciário.

Este estudo foi aprovado pelo Comitê de Ética em Pesquisa da Faculdade de Medicina de Botucatu da Universidade Estadual Paulista (protocolo CEP 4336-2012).

Foram incluídas todas as crianças de 1 a 12 anos de idade, ambos os sexos, com hipertrofia adenotonsilar e AOS, admitidas na UTIP após adenotonsilectomia, no período de janeiro de 2006 a dezembro de 2012. Foram excluídas crianças com anomalias cardíacas, pulmonares, neuromusculares ou cromossômicas e crianças com alterações craniofaciais, além de crianças que realizaram concomitantemente outros procedimentos cirúrgicos associados à adenotonsilectomia, tais como miringotomia, inserção de tubos de ventilação e/ou laringoscopia diagnóstica. As indicações de encaminhamento à UTIP se basearam em critérios clínicos e/ou polissonográficos, os quais incluíram: idade < 3 anos, obesidade, baixo peso, asma e/ou alterações polissonográficas (nadir da SpO_2_ ≤ 80%, IAH ≥ 10 eventos/h). 

Todos os pacientes foram avaliados por uma única pesquisadora durante o período perioperatório. Os parâmetros pré-operatórios foram coletados através de um questionário de anamnese padronizado que incluía idade, gênero, índice de massa corpórea (IMC), presença de comorbidades - obesidade (definida através das curvas do percentil de IMC para idade e gênero adotadas pela Organização Mundial da Saúde [2007], sendo percentil ≥ 97 critério de obesidade), asma, rinopatia e infecção de VAS (IVAS) - tamanho da adenoide (definido por um exame endoscópico com lente de nariz rígida 0° e 2,4 mm através da relação da adenoide com o espaço da rinofaringe) e tamanho da tonsila (estabelecido pelo escore de Brodsky).

A monitorização cardiorrespiratória para confirmar o diagnóstico de AOS foi realizada em regime de internação hospitalar, pela Disciplina de Otorrinolaringologia, por um período de até 6 meses antes da adenotonsilectomia. As crianças realizaram polissonografia tipo I (Alice^(r)^; Phillips Respironics, Murrysville, PA, EUA) ou polissonografia tipo III (Stardust II^(r)^; Phillips Respironics) ou oximetria de noite inteira para registro da SpO_2_ (PV 4000 LCD; Protec Equipamentos Médico-Hospitalares, São Paulo, Brasil). Os parâmetros respiratórios analisados foram: índice de apneia-hipopneia (IAH), índice de hipopneia (IH), índice de dessaturação de oxigênio (IDO) e nadir da SpO_2_. Os dados foram gravados por *software* específico de cada aparelho e pontuados por um único avaliador. A oximetria foi utilizada para definir o nadir da SpO_2_. A apneia obstrutiva foi definida por uma queda de mais de 90% no traçado da pressão nasal por pelo menos 2 ciclos respiratórios associada à presença do esforço torácico e/ou abdominal. A apneia central foi definida pela ausência de esforço inspiratório em todo o evento com duração ≥ 20 segundos ou duração de 2 ciclos respiratórios, associado a despertar ou dessaturação de O_2_ ≥ 3%. A hipopneia foi caracterizada por um decréscimo de pelo menos 50% no traçado da pressão nasal associado à dessaturação de O_2_ ≥ 3%. O IDO foi definido pelo número de episódios de dessaturação da oxi-hemoglobina ≥ 3% a partir da SpO_2_ basal por hora de sono com duração ≥ 10 segundos. O nadir da SpO_2_ foi definido como a mínima SpO_2_, independentemente da duração. A AOS foi classificada como leve (IAH de 1-4,9 eventos/h), moderada (IAH de 5-9,9 eventos/h) ou grave (IAH ≥ 10 eventos/h), e os eventos foram marcados segundo a pontuação da *American Academy of Sleep Medicine* (2007).

Todas as cirurgias foram supervisionadas, permitindo a padronização da técnica cirúrgica e a utilização de protocolo anestésico padronizado para crianças com AOS. Tanto a tonsilectomia como a adenoidectomia foram realizadas através de técnica fria convencional. A anestesia combinou a modalidade venosa (propofol [3mg/kg] e alfentanil [50mg/kg]) e inalatória (sevoflurano qsp e/ou N_2_O/O_2_ - 50/50). Após a cirurgia, todas as crianças foram encaminhadas intubadas à UTIP por um período de até 6 h e permaneceram na UTIP por período mínimo de 24 h, conforme protocolo institucional pré-estabelecido para crianças com apneia grave. As crianças foram divididas em dois grupos de acordo com a ausência ou presença de complicação respiratória após adenotonsilectomia.

As complicações respiratórias pós-operatórias foram divididas em maiores (SpO_2_ ≤ 80%, laringoespasmo, broncoespasmo, apneia, pneumonia [confirmada por radiografia de tórax], leucocitose com desvio à esquerda e febre, edema agudo de pulmão pós-obstrutivo [confirmado por radiografia de tórax ou uso de diuréticos de alça e reintubação]) e menores (SpO_2_ entre 90% e 80% com necessidade de reposicionamento de via aérea). Além disso, foram avaliados o tempo de intubação orotraqueal (IOT) após adenotonsilectomia e intervenções médicas (necessidade de reposicionamento de via aérea, uso de aparelhos de pressão positiva [*continuous positive airway pressure* ou *bilevel positive airway pressure*] e reintubação).

### Análise estatística

Para testar a hipótese de normalidade dos dados, aplicou-se o teste de Kolmogorov-Smirnov para todas as medidas contínuas dentro de cada grupo da amostra. O teste t de Student, o teste de Mann-Whitney e o teste do qui-quadrado foram utilizados para comparação intergrupos das variáveis estudadas. A análise de regressão linear múltipla foi realizada para determinar quais variáveis mais se relacionaram com o aumento do risco de complicações respiratórias após adenotonsilectomia.

Todos os testes foram realizados com o programa Statistica, versão 6.0 (StatSoft Inc., Tulsa, OK, EUA), adotando-se um nível de significância de 5%.

## Resultados

No período de janeiro de 2006 a dezembro de 2012 foram realizadas 805 adenotonsilectomias em crianças com AOS no hospital escola, e 53 dessas crianças foram encaminhadas à UTIP. Os motivos para encaminhá-las à UTIP foram idade < 2 anos (n = 2), obesidade e/ou asma com nadir da SpO_2_ ≤ 80% (n = 10), nadir da SpO_2_ ≤ 75% (n = 4), AOS leve com nadir da SpO_2_ ≤ 70% (n = 1), AOS moderada associada a comorbidades (n = 5) ou a nadir da SpO_2_ ≤ 80% (n = 2) e AOS grave (n = 29). Para o registro dos parâmetros respiratórios foram realizadas 7 polissonografias tipo I, 30 polissonografias tipo III e 6 oximetrias.

Dentre as comorbidades pesquisadas, o grupo com complicações apresentou maior frequência de rinopatia do que o grupo sem complicações (Tabela 1). O grupo com complicações respiratórias apresentou maior IAH, maior IDO, menor nadir da SpO_2_ e maior tempo de IOT em relação às crianças sem complicações (Tabela 2). Após análise de regressão linear múltipla, verificou-se que as seguintes variáveis independentes colaboraram para o aumento do risco de complicações respiratórias após adenotonsilectomia: IAH, IDO, nadir da SpO_2_, rinopatia e tempo de IOT [p(R^2)^ = 0,0099]; porém, individualmente, nenhuma variável demonstrou maior associação com o desfecho clínico (Tabela 3).

**Tabela 1.** Comparação dos dados demográficos e das comorbidades em crianças de acordo com a ausência ou presença de complicações respiratórias após adenotonsilectomia.a

Variáveis	Complicações respiratórias após adenotonsilectomia		p
	Ausência	Presença	
	(n = 32)	(n = 21)	
Masculino/Feminino	16/16	12/9	0,6062*
Idade, anos	6,2 ± 3,1	5,3 ± 2,6	0,2820**
< 3	4	3	0,4040***
3-6	13	10	
7-9	8	7	
10-12	7	1	
IMC, kg/m^2^	19,28 ± 5,27	18,76 ± 5,19	0,7257**
< percentil 3	0	2	0,8489***
≥ percentil 3 e < percentil 85	14	7	
≥ percentil 85 e < percentil 97	5	2	
≥ percentil 97	13	10	
Comorbidades			
Obesidade	13	10	0,4931*
Asma	4	6	0,1164*
Rinopatia^b^	20	25	0,0426*
IVAS vigente	0	2	0,0668*

IMC: índice de massa corpórea; e IVAS: infecção de vias aéreas superiores. aValores expressos em n de pacientes ou em média ± dp. bVariável selecionada para análise de regressão linear múltipla. *Teste do qui-quadrado; **teste t de Student; e ***teste de Mann-Whitney.

**Tabela 2.** Comparação dos dados polissonográficos, tempo de intubação orotraqueal e tamanho da adenoide e tonsilas em crianças de acordo com a ausência ou presença de complicações respiratórias após adenotonsilectomia.

Variáveis	Ausência de complicações		Presença de complicações		p
	n de pacientes	Média ± dp	n de pacientes	Média ± dp	
Variáveis polissonográficas					
IAH, eventos/h^a^	24	18,1 ± 11,2	13	28,6 ± 16,3	0,0269*
IH, eventos/h	24	5,8 ± 6,0	13	10,5 ± 10,4	0,0882*
IDO, episódios/h^a^	18	15,3 ± 9,4	12	29,8 ± 18,4	0,0082*
Nadir da SpO_2_,%^a^	28	76,8 ± 10,9	15	64,4 ± 16,9	0,0055*
Tempo de IOT no PO, h^a^	32	1,8 ± 2,0	21	5,0 ± 4,8	0,0011*
Tamanho de adenoide e tonsilas					
Adenoide, RAN	30	78 ± 17	17	87 ± 13	0,0705*
Tonsilas, escore de Brodsky					
1	0	−	0	−	0,3512**
2	4	−	2	−	
3	18	−	10	−	
4	10	−	9	−	

IAH: índice de apneia-hipopneia; IH: índice de hipopneia; IDO: índice de dessaturação de oxigênio; IOT: intubação orotraqueal; PO: pós-operatório; e RAN: relação adenoide/nasofaringe. aVariáveis selecionadas para análise de regressão linear múltipla. *Teste t de Student ; e **teste de Mann-Whitney.

**Tabela 3.** Análise de regressão linear múltipla considerando a complicação respiratória após adenotonsilectomia como variável dependente.a

Variáveis independentes	Ausência de complicações	Presença de complicações	Beta	Coeficiente parcial de regressão	Erro padrão	p	R^2^	p (R^2^)
IAH	18,1 ± 11,2	28,6 ± 16,3	−0,0276	−0,0009	0,0073	0,8972	0,3722	< 0,0099
IDO	15,3 ± 9,4	29,8 ± 18,4	0,2730	0,0082	0,0075	0,2798		
Nadir da SpO_2_	76,8 ± 10,9	64,4 ± 16,9	−0,0077	−0,0003	0,0062	0,9650		
Rinopatia	20	25	0,2679	0,3265	0,1843	0,0863		
Tempo de IOT no PO, h	1,8 ± 2,0	5,0 ± 4,8	0,3781	0,0527	0,0267	0,0568		

IAH: índice de apneia-hipopneia; IDO: índice de dessaturação de oxigênio; IOT: intubação orotraqueal; e PO: pós-operatório. aValores expressos em média ± dp ou em n de pacientes.

Das 53 crianças estudadas, 21 (39,6%) apresentaram complicações respiratórias após adenotonsilectomia. As crianças foram divididas segundo a ausência ou presença de complicações respiratórias (Tabela 1). O grupo sem complicações foi formado por 32 crianças, sendo 16 do gênero masculino e a idade média de 6,1 ± 3,1 anos (variação: 1,6-12). O grupo com complicações foi formado por 21 crianças, sendo 12 do gênero masculino e a idade média de 5,3 ± 2,6 anos (variação: 2,4-12). 

Sete crianças apresentaram complicações respiratórias menores (SpO_2_ entre 90-80%), e 14 crianças, complicações respiratórias maiores (SpO_2_ ≤ 80% [n = 2], laringoespasmos [n = 9], broncoespasmos [n = 5], broncoespasmo intraoperatório [n = 2], apneia [n = 1], pneumonia [n = 1] e edema agudo de pulmão [n = 3]; Tabela 4). O grupo com complicações respiratórias permaneceu na UTIP por um período ≥ 24 h, e as principais intervenções médicas foram antibioticoterapia (n = 1) por pneumonia, uso de diurético de alça (n = 3) por edema agudo de pulmão, nebulizações contínuas com broncodilatador ou adrenalina (n = 12) por broncoespasmos e laringoespasmos e reintubação (n = 3) por edema agudo de pulmão e broncoespasmo severo em vigência de IVAS (Tabela 4). Durante a evolução pós-operatória, a taxa de mortalidade foi zero, e as crianças com ausência e presença de complicações respiratórias permaneceram internadas por 3 ± 1 e 5 ± 2 dias, respectivamente.

**Tabela 4.** Resultados descritivos individuais de crianças com complicações respiratórias maiores e/ou menores após adenotonsilectomia.

Paciente	Sexo	Idade (anos)	Percentil do IMC	Complicações pós-operatórias	Reintubação	IAH	IH	IDO	< SpO_2_ (%)	Comorbidades
						(eventos/h)	(eventos/h)	(episódios/h)		
Complicações respiratórias maiores										
1	M	7,0	85-97	EAP	Sim	8,8	5,3	18,2	80	Asma + rinopatia
2	M	3,6	≥ 97	EAP + laringoespasmo	Sim	*	*	*	*	Asma + obesidade + rinopatia
3	M	3,3	≥ 97	EAP + laringoespasmo	Não	*	*	*	*	Obesidade + rinopatia
4	F	7,1	≥ 97	Laringoespasmo	Não	17,8	11,7	25,6	82	Obesidade + rinopatia
5	F	4,7	85-97	Laringoespasmo	Não	46,4	19	40,2	70	Rinopatia
6	M	7,7	≥ 97	Laringoespasmo	Não	*	*	*	*	Obesidade + rinopatia
7	M	2,6	50-85	Laringoespasmo + pneumonia	Não	*	*	*	42	IVAS + rinopatia
8	M	2,7	50-85	Laringoespasmo + broncoespasmo	Sim	*	*	*	72	IVAS + rinopatia
9	F	2,4	50-85	Laringoespasmo + broncoespasmo intra e pós-operatório	Não	*	*	*	*	Asma + rinopatia
10	F	3,3	50-85	Laringoespasmo + broncoespasmo	Não	18,7	7,2	16,6	55	Rinopatia
11	F	4,1	≥ 97	Broncoespasmo intraoperatório + SpO_2_≤ 80%	Não	*	*	*	*	Obesidade + rinopatia
12	M	3,2	50-85	Broncoespasmo + apneia	Não	33,1	2	30,8	63	Rinopatia
13	M	3,6	≥ 97	Broncoespasmo	Não	11,4	6,7	11,3	41	Asma + obesidade + rinopatia
14	F	3,6	15-50	SpO_2_ ≤ 80%	Não	*	*	*	*	Asma + rinopatia
Complicações respiratórias menores										
1	M	8,0	≤ 3	SpO_2_ entre 90-80%	Não	18,9	5,8	0	70	Asma + rinopatia
2	M	3,7	50-85	SpO_2_ entre 90-80%	Não	26,1	4,5	36,6	46	Asma + rinopatia
3	M	4,7	≥ 97	SpO_2_ entre 90-80%	Não	33,2	13,6	55,2	51	Obesidade
4	F	7,4	≥ 97	SpO_2_ entre 90-80%	Não	55,8	40,3	67,7	58	Obesidade + rinopatia
5	M	12,0	≤ 3	SpO_2_ entre 90-80%	Não	45,4	13,6	29,9	53	Rinopatia
6	F	8,0	≥ 97	SpO_2_ entre 90-80%	Não	48,3	7,2	23,8	88	Obesidade + rinopatia
7	F	7,2	≥ 97	SpO_2_ entre 90-80%	Não	8	0	1,9	95	Obesidade + rinopatia

IMC: índice da massa corpórea; IAH: índice de apneia-hipopneia; IH: índice de hipopneia; IDO: índice de dessaturação de oxigênio; EAP: edema agudo de pulmão; M: masculino; F: feminino; e IVAS: infecção de vias aéreas superiores.

As complicações respiratórias após adenotonsilectomia foram descritas individualmente na Tabela 4.

## Discussão

Das 805 crianças que realizaram adenotonsilectomia no período de janeiro de 2006 a dezembro de 2012 no hospital escola, 21 (2,6%) apresentaram complicações respiratórias pós-operatórias. Este dado se assemelha aos resultados de outros estudos, onde as complicações respiratórias variaram de 1,3% a 13,4%.^(^ 12 ^-^ 16 ^)^ Quando se avaliaram as crianças com alto risco para complicações encaminhadas à UTIP (n = 53), a taxa de complicações respiratórias foi de 39,6%, mostrando também correlação com outros trabalhos em que as taxas variaram de 25% a 60%.^(^ 11 ^,^ 17 ^-^ 21 ^) ^Vale ressaltar que nenhuma criança em acompanhamento pós-operatório de adenotonsilectomia na enfermaria pediátrica comum apresentou complicação respiratória maior com necessidade de transferência para a UTIP. 

O encaminhamento das 53 crianças à UTIP no período pós-operatório foi motivado pela presença de AOS associada a um ou mais fatores de risco. Pois de acordo com a literatura, crianças com AOS e/ou idade < 2 anos^(^ 9 ^,^ 11 ^,^ 17 ^,^ 18 ^)^ ou < 3 anos,^(^ 21 ^)^ obesidade,^(^ 9 ^,^ 12 ^,^ 15 ^,^ 22 ^-^ 24 ^)^ baixo peso,^(^ 9 ^,^ 21 ^)^ asma,^(^ 11 ^,^ 14 ^)^ alterações polissonográficas (nadir da SpO_2_ ≤ 80%^(^ 11 ^)^ ou < 72%,^(^ 9 ^,^ 15 ^,^ 19 ^)^ IAH ≥ 24 eventos/h,^(^ 17 ^)^ IH maior e/ou IAH maior),^(^ 15 ^)^ combinação de pressão de CO_2_ > 45 mmHg e SpO_2_ < 86%,^(^ 21 ^)^ presença de laringoespasmo intraoperatório^(^ 17 ^)^ e comorbidades sistêmicas associadas (anomalias neuromusculares,^(^ 9 ^,^ 11 ^,^ 20 ^)^ alterações craniofaciais,^(^ 9 ^,^ 11 ^,^ 20 ^)^ anomalias cardíacas^(^ 11 ^,^ 12 ^,^ 20 ^,^ 25 ^)^ e anomalias cromossômicas) ^(^ 11 ^,^ 12 ^)^ apresentam maiores índices de complicações respiratórias após adenotonsilectomia. Porém, após as análises dos resultados das complicações respiratórias pós-operatórias deste estudo, o serviço de Otorrinolaringologia da instituição não adota mais como rotina o encaminhamento à UTIP de pacientes < 12 anos com AOS e/ou fatores de risco após adenotonsilectomia. O que se preconiza é a realização de um estudo do sono para melhor caracterização da gravidade do distúrbio respiratório, além de minuciosa e detalhada história clínica para identificação de fatores de risco. Para pacientes com AOS grave associada a presença de comorbidades graves (anomalias cromossômicas, cardíacas, neuromusculares e alterações craniofaciais) e/ou crianças < 2 anos, ainda há a indicação de observação pós-operatória na UTIP.

Os fatores de risco capazes de predizer complicações respiratórias após adenotonsilectomia foram os parâmetros polissonográficos (maior IAH [média de 28,6 eventos/h], maior IDO [média de 29,8 episódios/h] e baixo nadir da SpO_2_ [média de 64,4%]), presença de rinopatia e maior tempo de IOT no pós-operatório (Tabela 2).

As complicações respiratórias são mais prevalentes em crianças quanto maior for o IAH, maior for o IDO e menor for o nadir da SpO_2_ no estudo do sono durante o pré-operatório. Nossos resultados foram similares aos encontrados em outros estudos que correlacionaram achados polissonográficos com complicações respiratórias pós-operatórias e intervenções médicas.^(^ 10 ^,^ 11 ^,^ 15 ^,^ 17 ^,^ 26 ^) ^Schroeder et al.^(^ 26 ^)^ observaram que 43% das crianças com IAH > 25 eventos/h necessitaram de alguma intervenção em decorrência de complicações respiratórias. Outro estudo mostrou que quanto maior o IAH (média de 31,8 eventos/h), maior o IH (média de 22,6 eventos/h) e menor o nadir da SpO_2_ (média de 71,7%), maior é a correlação com complicações respiratórias, sendo mais comumente as dessaturações que necessitaram de suplementação de O_2_.^(^ 15 ^)^ Portanto, é evidente a importância do conhecimento prévio da gravidade da apneia pelo estudo do sono (polissonografia) para predizer o risco de possíveis complicações respiratórias pós-operatórias. 

Dentre as comorbidades avaliadas, somente a rinopatia apresentou-se estatisticamente significante no grupo com complicações respiratórias. Como as crianças com AOS grave apresentam maior colapsabilidade das VAS^(^ 27 ^,^ 28 ^)^ e são mais suscetíveis aos efeitos respiratório-depressores das drogas anestésicas e dos opiáceos,^(^ 29 ^,^ 30 ^)^ é possível que a rinopatia associada ao aumento de secreções e edema das VAS possa contribuir para maior resistência e, consequentemente, um aumento de complicações respiratórias perioperatórias. 

Apesar de vários estudos na literatura demonstrarem que idade < 2 anos^(^ 9 ^,^ 11 ^,^ 17 ^,^ 18 ^)^ ou < 3 anos,^(^ 21 ^)^ obesidade^(^ 9 ^,^ 12 ^,^ 15 ^,^ 22 ^-^ 24 ^)^ e asma^(^ 11 ^,^ 14 ^)^ se relacionam com maior chance de complicações respiratórias após adenotonsilectomia, neste estudo não houve diferença estatisticamente significante para ocorrência de eventos respiratórios (Tabela 1). Pode ser que essas variáveis não se mostraram significantes por se tratar de uma amostra pequena, por este ser um grupo de crianças com maior risco de complicações respiratórias e pelo fato de as crianças apresentarem semelhança na distribuição das comorbidades.

O tempo de IOT (Tabela 2) foi estatisticamente maior em pacientes com complicações respiratórias, corroborando Schroeder Jr et al.,^(^ 26 ^)^ os quais demonstraram que retardar a extubação pode aumentar as chances de complicações respiratórias em um grupo que já possui alto risco. Portanto, as crianças que realizaram adenotonsilectomia para AOS associada a comorbidades e permaneceram intubadas após a cirurgia apresentaram maiores taxas de complicações e, consequentemente, maior tempo de internação hospitalar.

Este estudo foi limitado por uma amostra pequena, variedade na preferência do cirurgião para a observação pós-operatória e um possível viés na seleção das crianças que foram submetidas ao estudo do sono. Um estudo prospectivo com amostra maior se faz necessário para determinar os possíveis fatores de risco capazes de demonstrar uma maior chance de complicações respiratórias após adenotonsilectomia.^(^ 17 ^)^

Os resultados do presente estudo indicam que entre crianças com idade até 12 anos diagnosticadas com AOS, aquelas que têm maior IAH, maior IDO e menor nadir da SpO_2_ na avaliação polissonográfica pré-operatória, além da presença de rinopatia, possuem uma maior chance de desenvolver complicações respiratórias após adenotonsilectomia do que aquelas sem essas características.
